# Quantifying and resolving multiple vector transformants in *S. cerevisiae *plasmid libraries

**DOI:** 10.1186/1472-6750-9-95

**Published:** 2009-11-20

**Authors:** Thomas C Scanlon, Elizabeth C Gray, Karl E Griswold

**Affiliations:** 1 Thayer School of Engineering, Dartmouth College, Hanover, USA; 2 Department of Biological Sciences, Dartmouth College, Hanover, USA; 3 Molecular and Cellular Biology Program, Dartmouth College, Hanover, USA

## Abstract

**Background:**

In addition to providing the molecular machinery for transcription and translation, recombinant microbial expression hosts maintain the critical genotype-phenotype link that is essential for high throughput screening and recovery of proteins encoded by plasmid libraries. It is known that *Escherichia coli *cells can be simultaneously transformed with multiple unique plasmids and thusly complicate recombinant library screening experiments. As a result of their potential to yield misleading results, bacterial multiple vector transformants have been thoroughly characterized in previous model studies. In contrast to bacterial systems, there is little quantitative information available regarding multiple vector transformants in yeast. *Saccharomyces cerevisiae *is the most widely used eukaryotic platform for cell surface display, combinatorial protein engineering, and other recombinant library screens. In order to characterize the extent and nature of multiple vector transformants in this important host, plasmid-born gene libraries constructed by yeast homologous recombination were analyzed by DNA sequencing.

**Results:**

It was found that up to 90% of clones in yeast homologous recombination libraries may be multiple vector transformants, that on average these clones bear four or more unique mutant genes, and that these multiple vector cells persist as a significant proportion of library populations for greater than 24 hours during liquid outgrowth. Both vector concentration and vector to insert ratio influenced the library proportion of multiple vector transformants, but their population frequency was independent of transformation efficiency. Interestingly, the average number of plasmids born by multiple vector transformants did not vary with their library population proportion.

**Conclusion:**

These results highlight the potential for multiple vector transformants to dominate yeast libraries constructed by homologous recombination. The previously unrecognized prevalence and persistence of multiply transformed yeast cells have important implications for yeast library screens. The quantitative information described herein should increase awareness of this issue, and the rapid sequencing approach developed for these studies should be widely useful for identifying multiple vector transformants and avoiding complications associated with cells that have acquired more than one unique plasmid.

## Background

A key aspect of combinatorial protein engineering is the absolute requirement that each protein variant remain physically associated with its encoding gene (*i.e*. genotype-phenotype linkage). While there are numerous strategies for establishing and maintaining this critical link, the dominant approach remains recombinant microbial expression. The compartmentalized nature of microbial cells ensures the physical association of mutant genes with their cognate variant proteins, and also provides the molecular machinery and a regulated environment for efficient transcription and translation of library members.

Generally, it is assumed that transformation of microbial hosts with a recombinant plasmid library results in a "one cell - one mutant gene - one protein variant" paradigm. This assumption motivates clonal high throughput library screens as a strategy for identifying functionally enhanced proteins and their encoding genes. Although the first description of high efficiency *Escherichia coli *electroporation suggested that co-transformation with multiple plasmids may occur at high DNA concentrations [1], the potential for simultaneous transformation of more than one plasmid into any given microbial cell during library construction has typically been dismissed as an improbable event [2]. Furthermore, it has been assumed that rare occurrences of multiple vector transformants (MVT ≡ a host bearing two or more plasmids distinguished only by the sequences of their respective mutant genes) are quickly resolved into clonal progeny by plasmid partitioning during cell division [3,4]. Thus, protein engineers have long been comfortable in the assumption that sequenced genes from selected clones do in fact encode for the proteins producing the observed clonal phenotype. While the one cell - one mutant gene - one protein variant paradigm has proven sufficiently accurate in the vast majority of reported experimental outcomes, there have been several high profile retractions wherein confounding results and inaccurate conclusions were specifically traced to contamination from MVT [5,6] and other cases in which MVT could have contributed to unexplained *in vivo *results [7]. These examples underscore the liabilities associated with MVT in recombinant library populations.

To examine the issue of MVT in bacterial libraries, 2 and 3-plasmid model systems based on orthogonal selectable markers have been characterized in detail. One report demonstrated that multiply transformed cells can represent up to 0.4% of electroporated *E. coli *populations under the conditions tested [2]. A second study showed that MVT resulting from phagmid infection can persist as a significant proportion of cell populations even after five consecutive overnight outgrowths [8]. These reports have significantly advanced knowledge regarding MVT in the Gram-negative bacterial host *E. coli*, but no analogous detailed information regarding yeast libraries is currently available.

Early studies of *Saccharomyces cerevisiae *plasmid transformation indicated that this common yeast host may exhibit a higher than expected frequency of MVT [9], although these preliminary studies involved low efficiency spheroplast-CaCl_2 _transformation, which is not suitable for construction of large libraries. Subsequent studies using low copy number plasmids bearing centromeric origins of replication revealed toxicity associated with MVT [10], suggesting that their persistence was unlikely. Thus, while it has long been known that *S. cerevisiae *are prone to acquisition of multiple plasmids during transformation, there remains a dearth of quantitative information describing the prevalence and maintenance of MVT events in conditions representative of recombinant library screening experiments. Furthermore, no previous studies have addressed methods for reducing the occurrence of MVT. Given the widespread use of this microorganism as a screening platform for combinatorial protein engineering, two hybrid protein-protein interactome mapping, and other library screening protocols, a detailed characterization of *S. cerevisiae *MVT was undertaken in the context of low copy number plasmid libraries constructed by yeast mediated homologous recombination. Experiments were designed to answer three key questions regarding the nature of MVT in recombinant *S. cerevisiae *libraries:

1) What fraction of successfully transformed library members bear two or more plasmids?

2) Among these MVT, how many unique mutant genes does the average clone harbor?

3) Under standard liquid growth conditions, how long do MVT persist as a significant proportion of the library population?

The answers to these questions have wide ranging implications for laboratories using yeast as an expression and screening platform for recombinant DNA libraries.

## Results

### Proportion of multiple vector transformants in initial library populations

Previous studies using a selection based, two plasmid model system in *E. coli *demonstrated that transformation of this bacterial host with 500 ng of plasmid DNA resulted in a 0.4% double transformant frequency [2]. Note that these experiments were designed to model parental plasmid contamination and therefore used a skewed 1:1250 ratio of the two model plasmids. To assess the proportion of MVT in yeast libraries generated using similar total plasmid concentrations, site-directed lysozyme gene libraries were constructed using the ISOR strategy [11], and freshly prepared electrocompetent *S. cerevisiae *were transformed with 500 ng of vector backbone and 1.25 μg of gene insert yielding library L-1:2.5. Control transformations with linear vector alone yielded approximately 1000-fold fewer transformants than did the transformations with both vector and gene insert (data not shown). In the studies presented below, all mutations mapped to one of eight codons that were specifically targeted by combinatorial mutagenesis. This observation provides strong evidence that the sequenced mutations resulted from library construction and not from downstream PCR processing.

**Figure 1:**
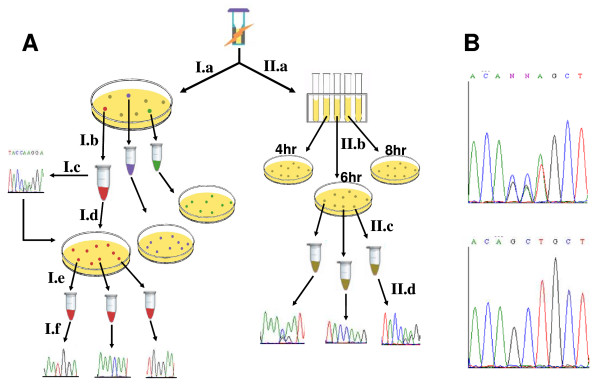
**Workflow and sequencing method for library analysis**. (A) Transformed libraries were handled one of two ways: (I) Sample proportions of MVT were assessed by plating serial dilutions on selective media yielding resolved colonies upon outgrowth (I.a). Genes from ~20 colonies were sequenced using an optimized, cell-templated protocol (I.b and I.c), and MVT were identified by overlapping peaks in their sequencing chromatograms. To estimate the average number of unique plasmids born by these MVT, ten verified MVT were restreaked to single cell resolution on fresh selective plates (I.d). Genes from ~10 colonies of each restreaked plate were sequenced (I.e and I.f), and the number of plasmids in the corresponding parental clone was estimated from the number of unique genes per plate. (II) In parallel experiments, the library proportion of MVT was assessed as function of outgrowth time. Following transformation, cells were grown with aeration in selective liquid media at 30°C (II.a). Every two hours, aliquots were plated yielding resolved colonies upon outgrowth (II.b). Eleven or more colonies from selected time points were assessed for the presence of multiple plasmids by DNA sequencing (II.c and II.d). (B) Top - chromatogram from a MVT colony exhibiting overlapping peaks (target bases 121-124). Note that the ABI base calling algorithm incorrectly identifies the third multiple peak as an "A". This underscores the importance of visual chromatogram inspections. Bottom - corresponding chromatogram from a monoclonal colony template.

To assess the frequency of MVT in L-1:2.5, an aliquot of the transformation mixture was diluted and plated on selective media at a concentration appropriate to yield spatially segregated, spherical colonies upon outgrowth. The mutant gene(s) encoded by each yeast colony were amplified using an optimized procedure for yeast-templated, high fidelity PCR as described in the materials and methods (see Figure 1A for complete workflow). The resulting PCR amplicons from 19 randomly selected colonies were subsequently sequenced.

**Figure 2:**
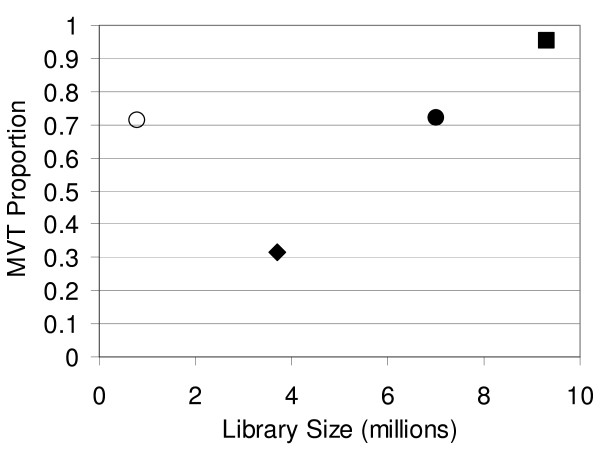
**Sample proportion of MVT in various libraries**. Library proportion of MVT plotted as a function of library size. Open Circle, H-1:2.5*; Closed Diamond, L-1:2.5; Closed Circle, H-1:2.5; Closed Square, H-1:5.

Based on visual inspection of the sequencing chromatograms (Figure 1B), greater than 30% of the sampled colonies were MVT (Figure 2). While the estimated 0.35 population proportion of MVT in library L-1:2.5 (Table 1) was almost 100-fold greater than that previously reported for *E. coli*, the DNA concentration in this transformation solution was relatively low compared to typical transformation protocols designed to yield large yeast libraries.

**Table 1:** Library properties and statistics

Library	Library Construction Details			Population Proportion of MVT		Unique Plasmids per MVT	
	Vector (μg)	Insert (μg)	Size (cfu ×10^6^)	Wilson Estimate	95% CI	Median Unique Genes per MVT	95% CI
L-1:2.5	0.5	1.25	3.7	0.35	0.15 < p < 0.54	N.D.	N.D.
H-1:2.5	1	2.5	7.0	0.69	0.52 < p < 0.86	4.9	4.5 to 7.5
H-1:2.5*	1	2.5	0.8	0.68	0.50 < p < 0.86	N.D.	N.D.
H-1:5	1	5	9.3	0.88	0.76 < p < 1.00	3.9	2.6 to 7.0

* Library generated using low efficiency, freeze-thawed electrocompetent cells.

To assess the frequency of MVT in an *S. cerevisiae *library generated using a standard protocol [12], freshly prepared electrocompetent yeast were transformed with 1 μg of vector backbone and 5 μg of mutagenized DNA insert yielding library H-1:5. Following DNA sequence analysis of 21 randomly selected colonies, the observed sample proportion of MVT was 0.95 (Figure 2), and the estimated population proportion was 0.88 (Table 1). The difference between MVT proportions in H-1:5 and L-1:2.5 was clearly significant (P < 0.0005), but it was unclear whether the increased MVT frequency in H-1:5 was a result of the 2-fold increased concentration of vector backbone, the 2-fold increased ratio of insert to vector, or some combination thereof. Therefore, a third library (H-1:2.5) was generated by transforming freshly prepared yeast with 1 μg of vector backbone (same vector mass as H-1:5) and 2.5 μg of gene insert (same vector:insert ratio as L-1:2.5). A total of 18 MVT were found amongst 25 randomly sampled colonies (Figure 2), and the estimated H-1:2.5 population proportion of MVT was 0.69 (Table 1). This proportion was significantly different from that of L-1:2.5 (P = 0.008) and H-1:5 (P = 0.038). These results suggest that both vector backbone concentration as well as gene insert to vector ratio are determinants of yeast MVT frequency.

A closer inspection of the above results suggested a linear correlation between MVT frequency and library size (Figure 2, closed symbols). To determine if MVT frequency did in fact exhibit a simple correlation with transformation efficiency, library H-1:2.5 was remade by low efficiency transformation of freeze-thawed electrocompetent yeast yielding library H-1:2.5*. While the size of H-1:2.5* was 10-fold smaller than H-1:2.5, the proportion of MVT in the small freeze-thaw library was essentially identical to that of the corresponding larger library (P = 0.966, Table 1). Thus, while DNA concentration plays a critical role determining library proportion of MVT (see above), it appears that MVT proportions are independent of overall transformation efficiency (Figure 2, note open circle).

### Average number of unique genes in multiple vector transformants

In addition to knowing the fraction of MVT in a library population, it would also be advantageous to know the average number of unique plasmids harbored by multiply transformed cells. This information is particularly relevant given the dominance of MVT in the 1 μg vector backbone libraries. To estimate this value, aqueous suspensions of 10 randomly selected MVT from both library H-1:5 and H-1:2.5 were restreaked to single cell resolution on dextrose growth agar yielding isolated, spherical, progeny colonies upon outgrowth (Figure 1A). Lysozyme genes from 9 to 13 individual colonies of each plate were sequenced (2 libraries • 10 MVT per library • ~10 colonies per MVT ≈ 200 sequences). The number of unique gene sequences harbored by each MVT was extrapolated from these data using a first order jackknife estimator [13], and the population median for each library was derived from the distribution of values for the 10 corresponding clones (Table 1). MVT from library H-1:5 were estimated to harbor a median of 3.9 unique plasmids and those of H-1:2.5 carried 4.9 unique plasmids. Because data from H-1:2.5 did not appear to be normally distributed (data not shown), a non-parametric Wilcoxon-Mann-Whitney test was used to determine that the distributions of unique plasmids among MVT of H-1:2.5 and H-1:5 were not significantly different (P = 0.16). Thus it is interesting to note that, while library proportions of MVT appear to be a function of gene insert concentration (see above), the average plasmid content of MVT populations is independent of this variable.

### Persistence of multiple vector transformants during liquid culture outgrowth

**Figure 3:**
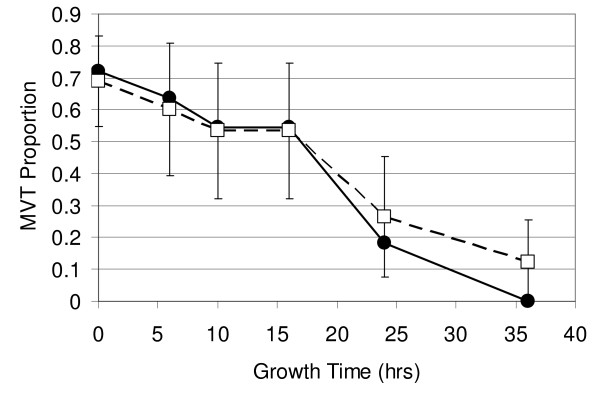
**MVT as a function of growth time**. Library H-1:2.5. Closed circles - observed sample proportions; Open squares - Wilson estimate of population proportions; Error bars - 95% confidence intervals for population proportions. Lines are provided as a visual guide only.

One approach to eliminate MVT from library populations is to perform liquid culture outgrowth leading to segregation and copy number control of CEN-based plasmids during cell division [14]. In an effort to determine the time necessary to eliminate MVT as a significant subpopulation, their frequency in the H-1:2.5 library was assessed as a function of growth time. Following electroporation, the transformed library was subcultured into dextrose growth media and grown with aeration at 30°C for up to 36 hours. Cultures were serial diluted to prevent saturation during outgrowth. Aliquots were spread to single cell resolution on dextrose growth agar every two hours for the first 24 hours. An additional aliquot was plated at 36 hours. Following incubation of the plates to yield spatially resolved single colonies, 10-12 randomly selected spherical colonies from various time points were sequenced as described in the materials and methods. The observed proportion of MVT in the library population was plotted as a function of outgrowth time (Figure 3). Surprisingly, 20% of the sampled clones continued to harbor more than one unique plasmid even after 24 hours of outgrowth. Not until the 36 hour time point did the MVT subpopulation decrease below the detection threshold for the experimental sample size.

## Discussion

While the prevalence and persistence of MVT in recombinant *E. coli *libraries has been studied previously, the issue of MVT in *S. cerevisiae *libraries has received only cursory and indirect attention. To shed light on this important issue, MVT were quantitatively analyzed in experimental libraries constructed by yeast homologous recombination. Unlike model systems based on selective genetic markers that produce readily observed phenotypes (*e.g*. growth or no growth), the practical nature of the libraries studied here necessitated direct DNA sequencing to identify and characterize MVT. As such, the sample sizes used in this study are smaller than those that would have been possible using orthogonal antibiotic or auxotrophic markers as a model system. However, analysis of a realistic library, currently in use for practical protein engineering, provided insights that would have been inaccessible using any convenient model. For example, an estimation of average unique plasmids harbored by MVT cells would not be accessible *a priori *using a model system with a small number of selectable markers; an accurate estimate requires that the mean number of unique sequences per MVT be exceeded by the total number of unique sequences in the library population. Furthermore, due to the particularly high degree of similarity among genes in mutant libraries, yeast mediated homologous recombination between two or more unique genes in any given MVT could produce additional library diversity not present in the original transformation mixture [15]. The lack of similarity between genes for different antibiotic or auxotrophic selection markers dictates that such post-transformation diversification would not be captured by convenient model systems. Therefore, analysis of an actual mutant gene library was employed in order to provide data with more direct relevance to practical library screening experiments.

Underscoring the potential impact of yeast MVT, it was observed that cells bearing multiple plasmids persisted in library populations at ~20% frequency for more than 24 hours, despite the stringently regulated low copy CEN/ARSH4 origin employed here. Given the potential for MVT to interfere with accurate interpretation of experimental results, the staggering proportions of MVT observed in these studies advocates for careful examination of this issue as a part of any yeast library screening protocol. It is emphasized that the studies described here employed a plasmid bearing the URA3 selectable marker, and alternative markers were not examined. In previous studies, however, CEN6/ARSH4 plasmids bearing either the URA3, LEU2, HIS3 or TRP1 markers were shown to exhibit less than a 2-fold difference in transformation efficiency, and the mitotic stabilities of these markers were similar under selective pressure [16]. These results suggest that a plasmid's auxotrophic marker would not be a dominant factor relating to MVT in library populations.

Our results suggest two alternative strategies for limiting MVT in recombinant *S. cerevisiae *libraries. In one approach, *S. cerevisiae *libraries can be outgrown for extended time periods, i.e. > 24 hours, in media repressing recombinant gene expression. Importantly, it is possible that yeast vectors bearing higher copy number origins, for example the 2 μm origin, would require even longer outgrowths for complete segregation of plasmids to meet the one cell-one mutant gene-one protein variant paradigm. As an alternative to long outgrowths, lower concentrations of DNA may be used to transform yeast hosts thereby reducing the frequency of MVT. Compare for example the fraction of MVT in libraries L-1:2.5 and H-1:2.5 (Figure 2). However, the benefit of reduced MVT frequency must be weighed against the smaller size of libraries obtained with lower DNA concentrations.

Although the hypercompetent nature of *S. cerevisiae *represents a potential liability for clonal library screening, it may also constitute a previously unrecognized advantage to the protein engineer. The size of library populations is typically determined by plating serial dilutions of transformed cells onto selective media shortly after the transformation procedure. Colony forming units are enumerated following outgrowth, and the total library size is back calculated. Based on the results reported here, this established methodology may under represent the true diversity of analogously constructed yeast libraries by 4-fold or more. This additional diversity may be accessed by allowing sufficiently long outgrowths to completely segregate MVT plasmids prior to clonal library screening. Alternatively, a single cell variation of "pooled" clone screening [17] could be implemented to take advantage of the additional diversity. From this perspective, MVT in a library represent a diversity advantage, provided that one is fully aware of the potential pitfalls and remedies for addressing them.

Ultimately, great care must be taken by the protein engineer to ensure that any observed improvement in protein function is correlated with the correct mutant gene sequence. Conventional validation of the genotype-phenotype link in selected *S. cerevisiae *library members requires outgrowth of the isolated yeast cells, isolation of low quality/quantity yeast plasmid DNA, transformation into an *E. coli *host, outgrowth of the transformed bacteria on solid media, outgrowth of selected bacterial transformants in liquid media, isolation of high quality/quantity plasmid DNA from bacterial cells, sequencing gene insert from several colonies, retransformation of the sequenced plasmid into the yeast host, and revalidation of the originally observed phenotype. Due to slow growth rates of yeast, this process typically results in inconveniently long timeframes for clone validation (>1 week), and it is particularly arduous when validating large numbers of clones. This time, labor, and resource intensive process limits productivity, and can easily become intractable for small labs examining large libraries.

In contrast to the conventional, laborious, shuttle vector strategy described above, the yeast-templated PCR and direct amplicon sequencing approach developed for these studies yields robust sequencing results in roughly 1 day. Importantly, this technique was shown to be highly reliable as verified by extensive characterization of a combinatorial, site-directed mutant library in which all sequenced mutations mapped to the target sites. It is anticipated that other researchers may find this procedure useful in speeding analysis and preliminary validation of clones selected from recombinant yeast libraries, although genes of particular interest should ultimately be confirmed *via *sequencing of high quality plasmid template.

## Conclusion

Using carefully designed DNA sequencing studies of a site-directed mutant gene library, the frequency and persistence of MVT in *S. cerevisiae *has been quantitatively evaluated with CEN/ARSH4 based plasmids bearing a URA3 selectable marker. In libraries constructed by electroporation and homologous recombination, both the vector backbone concentration and the vector to gene insert ratio were found to influence the population proportion of MVT. However, MVT frequency was shown to be independent of transformation efficiency. An awareness of the potential for MVT to dominate yeast libraries will help researchers avoid associated pitfalls. To facilitate rapid validation of genotype-phenotype linkage in yeast clones selected from recombinant libraries, an optimized procedure for yeast-templated, high fidelity PCR and direct amplicon sequencing has been described.

## Methods

### Reagents and cells

Oligonucleotides for sequencing and mutagenesis (50 nmol scale, desalted) and oligonucleotides for yeast homologous recombination (50 nmol scale, HPLC purified) were purchased from Integrated DNA Technologies (Coralville, IA). Plasmids, bacteria, and yeast were obtained from the American Type Culture Collection (Manassas, VA). Restriction enzymes, modification enzymes, dNTP's, and polymerase were from New England Biolabs (Beverly, MA). Growth media was from BD Diagnostics (Sparks, MA), except CSM-ura supplements which were from MP Biomedicals (Cleveland, OH). Guanosine was purchased from Sigma-Aldrich (St. Louis, MO). Zymolyase, gel extraction, and DNA clean up kits were obtained from Zymo Research (Orange, CA). Unless specifically noted, all other reagents were purchased from Fisher Scientific (Pittsburgh, PA).

### Construction of the human lysozyme (LYZ) expression vector

The yeast αMF leader sequence with Kex2 processing site was amplified from the pGAPZαA vector (Invitrogen) via PCR with Phusion Polymerase using oligonucleotides appending the sequences for 5'-Spe1 and 3'-EcoRV restriction sites (Spe1_GAPAα: 5'-GTCATACTAGTATGAGATTTCCTTCAATTTTT-3'; GAPZα_EcoRV: 5'-GTCATGA TATCCCGAGACGGCCGGCTGGGCCA-3'). The PCR amplicon was purified by 1% agarose gel electrophoresis and Zymoclean Gel DNA Recovery Kit. Subsequently, 1 μg was digested with Spe1 and EcoRV. Plasmid p416-GAL1 [18] was digested with XhoI, blunt-ended with Klenow Polymerase followed by heat-inactivation and digestion with Spe1 creating ends compatible with the digested αMF amplicon. The digested amplicon and vector were ligated using T4 Ligase and transformed into E. coli DH5α [F-ϕ80dlacZΔM15 Δ(lacIZYA-argF)U169 deoR *rec*A1 *end*A1 *hsd*R17(r_k_^-^, m_k_^+^) *sup*E44 *thi*-1 *gyr*A96 *rel*A1 λ-] yielding plasmid p4GM. Plasmid pOTB7, containing the coding sequence for human lysozyme (lyz) was obtained from ATCC (I.M.A.G.E.: 2959387). The coding sequence (390 bp) was amplified via PCR with Phusion Polymerase appending a 5'-sequence encoding a Kex2 processing site with XhoI restriction site and a 3'-EcoRI restriction site (Xho1_LYZ: 5'-TCTCTCGAGAAAAGAAAGGTCTTTGAAAGGTGTGAG-3'; LYZ_EcoR1: 5'-GTCATGAATTCTTATTACACTCCACAACCTTGAACATACTG-3'). The PCR amplicon was purified by 1% agarose gel electrophoresis and Zymoclean Gel DNA Recovery Kit. It was subsequently digested with XhoI and EcoRI, and ligated with similarly digested p4GM vector creating vector p4GM-LYZ, coding for the wild type lyz gene in frame with the α-factor signal sequence and Kex2 signal processing site.

### Gene library construction

The coding sequence of the entire α-factor signal sequence and lyz gene was amplified in 8 × 100 uL PCR reactions with Phusion polymerase and external primers (GAPseqA1 5'-CCAACAGCACAAATAACGGG-3'; CYC1TseqB1 5'-TCGGTTAGAGCGGATGTGGG-3'), PCR purified over 6 Zymo Spin1 DNA purification columns, and eluted with deionized water yielding a final DNA mass of 23 μg. Exactly 5 μg of lyz amplicon was diluted into 50 μL of 1XDNaseI reaction buffer (NEB), and equilibrated for 2 minutes at 15°C in a DNA Engine thermocycler (Bio Rad). Two units of DNaseI were added to the chilled solution, and the digestion reaction was incubated for 2 minutes at 15°C. Subsequently, the entire reaction contents were transferred to 110 μL of Zymo DNA Binding Buffer to inactivate the DNase. The contents of the reaction were purified over a Zymo Spin1 DNA purification column, and eluted with 10 μL of TE. The entire contents were subjected to gel electrophoresis in 2% agarose containing 1 mM guanosine, and reaction products in the size range of 50-150 bp were purified from the gel using Zymoclean Gel DNA Recovery Kit.

Gene reassembly using size-selected DNA fragments and mutagenic synthetic oligonucleotides was performed essentially as described [11]. Three mutagenic oligonucleotides were targeted to each of eight LYZ sites resulting in the combinatorial mutation of these residues to each of 3 alternative amino acids (4 possible codons per site, including the wild type sequence). Each mutagenic oligonucleotide contained 15 base pairs of complimentarity to lyz cDNA on either side of the target codon; the sequence of mutant codons was selected based on preferred *S. cerevisiae *codon usage. The gene reassembly reaction was performed in 50 μL of 1XHF Phusion Polymerase buffer containing 200 μM dNTP, 2U Phusion polymerase, 100 ng size-selected lyz amplicon, and a mix of 24 mutagenic oligonucleotides, each at a final concentration of 300 nM. The thermocycling program was comprised of an initial denaturing step of 98°C for 30 seconds, followed by 25 cycles of the following progression: 98°C for 10 seconds, 65°C, 62°C, 59°C, 56°C, 53°C, 50°C, 47°C, 44°C, 41°C for 30 seconds each, and 72°C for 30 seconds. A final elongation step of 72°C for 5 minutes was performed for maximal production of full-length reaction products. The reassembly reaction was diluted 20 fold in deionized water and stored at -20°C until further use. A nested PCR reaction was used to generate a quantity of full-length mutant lyz amplicon sufficient for yeast transformation. Briefly, 5 × 100 μL reactions were performed in 1XHF Phusion Polymerase buffer containing 150 μM dNTP, 2U Phusion polymerase, 1 μM terminal oligonucletides as priming agents, and 3 μL of the diluted reassembly reaction per 100 μL reaction. The nested PCR reactions were pooled and purified via Zymo SpinI DNA column purification. Typical yield from the nested PCR reaction was 3-5 μg per 500 μL reaction.

### Preparation of electrocompetent *S. cerevisiae*

A single colony from a freshly-streaked plate of protease-deficient *S. cerevisiae *strain BJ5464 [MATalpha *ura*3-52 *trp*1 *leu*2-delta1 *his*3-delta200 *pep4*::HIS3 *prb*1-delta1.6R *can*1 GAL] was inoculated into 500 mL of YPD, and grown at 30°C with shaking at 250 rpm on a rotary platform (1" orbit) in a 2.0 L baffled flask. Yeast were grown until the cell density reached an OD600 nm 1.0 (~20 hours), and immediately chilled in an ice water bath for 15 minutes to slow cell growth. The yeast were pelleted by centrifugation in a fixed-angle centrifuge at 4500 *g *for 5 minutes at 4°C. The pellet was resuspended in 50 mL of electrocompetent buffer (100 mM Lithium Acetate, 10 mM DTT, 1.0 mM EDTA, 10 mM TrisHCl, pH 7.5) and gently shaken on a rotary platform at 80 rpm for 1 hour at 30°C. Following this treatment, 450 mL of ice cold deionized water was added to the suspension, and the yeast were centrifuged at 4500 *g *for 5 minutes at 4°C. The pellet was resuspended in 250 mL ice cold deionized water and centrifuged as above. Yeast cell pellet was resuspended in 20 mL ice cold 1 M sorbitol, transferred to 50 mL conical tubes, and immediately centrifuged at 2800 *g *in a swinging bucket centrifuge (Allegra 6R, Beckman Coulter) for 10 minutes at 4°C. Cell pellets were resuspended in 350 μL ice cold 1 M sorbitol and immediately aliquoted into prechilled 1.5 mL eppendorf tubes in 40 μL aliquots. Freshly prepared electrocompetent yeast were used for most library transformations, and the remainder was frozen at -80°C for use in low efficiency transformations.

### Yeast transformation

The library of mutagenized lyz amplicons was inserted into the p4GM expression vector via yeast gap repair homologous recombination [19]. Briefly, the vector p4GM-LYZ was digested with XhoI and EcoRI to excise the entire wild type lyz coding sequence. The digestion reaction was purified with a Zymo DNA Clean and Concentrator Kit. Note: in control experiments it was found that the number of background transformants derived from agarose gel purified vector was identical to that derived from Zymo DNA Clean and Concentrator purified vector (data not shown). Thus the restriction enzyme excised lyz gene was unable to efficiently recombine with the digested vector backbone upon transformation into *S. cerevisiae*, and gel purification of the digested vector was deemed unnecessary. Nested PCR amplicon from the reassembly reaction contains > 40 base pairs of flanking homology to the digested p4GM plasmid backbone, and is therefore suitable for yeast gap repair without further modification. Various quantities of digested plasmid backbone DNA and nested PCR amplicon (see Table 1) were combined with a 40 μL aliquot of electrocompetent BJ5464, and chilled on ice for 5 minutes. The mix was transferred to a prechilled 0.2 cm gap electroporation cuvette (Fisher Scientific). Electroporation was performed with the following settings: voltage 1100V, capacitance 25 μF, Pulse Controller set to 200 ohm. Immediately following electroporation, the cells were diluted with 1.0 mL ice cold 1 M sorbitol and allowed to stand at 25°C for 5 minutes. Subsequently, aliquots were either subcultured into selective dextrose growth media (0.67% yeast nitrogen base, 0.77 g/L CSM -ura, 2% dextrose, 100 mM KHPO4, pH 6.0) or plated on selective dextrose growth agar (dextrose growth media with 1.5% agar and 1 M sorbitol) and grown at 30°C. Vector only control transformations were done in parallel with the library transformations.

### Identification of multiple vector transformants

Immediately following electroporation and recovery, diluted cells from each transformation reaction were plated on dextrose growth agar at a density appropriate to yield spatially separated colonies (see Figure 1A for complete workflow). Following 36 hours of outgrowth on the transformation plate, randomly selected, spherical colonies were individually harvested with sterile filter pipet tips, resuspended in sterile deionized H_2_O, restreaked to single cell resolution on fresh dextrose growth media ("restreak plate", see below), and an aliquot of the aqueous suspension was diluted to 20 μl in digestion buffer (50 mM Tris, 5 mM EDTA, pH 7.8). Cell suspensions in digestion buffer were combined with 4U of Zymolyase (Zymo Research), incubated at 37°C for 4 hours, and then stored at 4°C until ready for use. Digested yeast cell suspensions were diluted 1:1 with 40 mM NaOH, and cells were lysed by heating at 99°C for 10 minutes. The cell lysate (1.25 ul) was used immediately as template in a 25 ul PCR reaction containing 1XPhusion HF buffer, 200 uM each dNTP, 1 uM of each external primer (pGAPseqA1 and CYC1TseqB1), and 0.5 units of Phusion Polymerase. Negative control reactions lacking the yeast cell lysate were run using the same reagent master mix. The thermocycling profile consisted of an initial melting step at 98°C for 30 sec, followed by 30 cycles of 98°C-6 sec; 60°C-20 sec; 72°C-15 sec. A final 5 minute extension at 72°C was included. Completed reactions were run on a 1% agarose gel containing 1 mM guanosine, the product band at 548 bp was excised, and the DNA was purified with a Zymoclean Gel DNA Recovery Kit. Negative control reactions uniformly failed to yield any detectable PCR product. The gel purified PCR amplicons were sequenced using a dideoxy terminator protocol on an ABI Model 3100 genetic analyzer. The resulting chromatograms were visually inspected, and colonies were designated as MVT if one or more of the eight target sites showed multiple peaks (Figure 1B).

### Quantifying unique plasmids in multiple vector transformants

Clones identified as MVT (see above) were used to estimate the number of unique plasmids born by the respective parental clone. Briefly, parental MVT colonies were restreaked on dextrose growth agar, and incubated at 30°C for 36 hours. Importantly, the spatially separated colonies on each restreaked plate represent clonal populations of cells derived from a single MVT. Ten randomly selected clones from each restreaked plate were harvested and sequenced as outlined above (Figure 1A). The resulting sequencing chromatograms were visually inspected to verify that each sequence represented a single mutant gene, and the number of unique genes per restreaked plate was tallied and used to estimate the number of plasmids in the original parental MVT.

### Evaluating persistence of multiple vector transformants

Following electroporation, an aliquot of library H-1:2.5 was serial diluted 1:10, 1:100, 1:1000, and 1:10000 into dextrose growth media. Three ml of each dilution was aliquoted in quadruplicate into sterile 18 × 150 mm culture tubes, and the suspensions were incubated with aeration at 30°C. At regular intervals following transformation (2, 4, 6, 8, 10, 12, 14, 16, 18, 20, 22, 24, and 36 hours), one tube of cells not yet nearing saturation was withdrawn, and a small aliquot was streaked to single cell resolution on dextrose growth agar. The plate was subsequently grown 36 hours at 30°C resulting in formation of spatially separated colonies. Colonies were assessed for the presence of multiple plasmids by DNA sequencing as outlined above.

### Statistical analysis

Statistical analysis was done in Minitab (Minitab Inc). Given the small sample sizes in these studies, library proportions of MVT and the 95% confidence intervals for these proportions were estimated (Wilson Estimate) using the Adjusted Wald Method [20]. A χ2 test was used to compare the proportion of MVT from various libraries (null hypothesis, H0: the MVT proportion of the first library is equal to the MVT proportion of the second library). The number of unique plasmids in each of 10 randomly selected MVT from libraries H-1:2.5 and H-1:5 was derived from a 1st order jackknife estimator [13] using sequencing results from 9 to 13 individual progeny of each parental MVT. The distribution of these estimates was used to calculate the 95% confidence interval for median unique plasmids born by MVT in each library population. Differences in population distribution of unique plasmids per MVT in libraries H-1:2.5 and H-1:5 were analyzed with a Wilcoxon-Mann-Whitney test [21].

## Authors' contributions

KG and TS conceptualized the project and designed all experiments. TS constructed the recombinant gene libraries. EG and KG transformed the libraries into yeast hosts, and prepared samples for sequencing. EG performed the sequence analysis. KG performed the statistical analysis. KG and TS prepared the manuscript. All authors have read and approved the final manuscript.
